# Ten‐year outcomes of hip arthroscopy for femoroacetabular impingement with osteoarthritis: Sustained functional benefits but high conversion to total hip arthroplasty

**DOI:** 10.1002/ksa.12709

**Published:** 2025-05-27

**Authors:** Sarantos Nikou, Carl Sandlund, Ida Lindman, Per‐Erik Johansson, Axel Öhlin, Louise Karlsson, Mikael Sansone

**Affiliations:** ^1^ Department of Orthopaedics, Institute of Clinical Sciences, Sahlgrenska Academy University of Gothenburg Gothenburg Sweden; ^2^ General Practice/Family Medicine, School of Public Health and Community Medicine, Institute of Medicine, Sahlgrenska Academy University of Gothenburg Gothenburg Sweden; ^3^ Research, Education, Development & Innovation Primary Health Care Västra Götaland Sweden; ^4^ Department of Orthopaedic Surgery South Älvsborg Hospital Borås Sweden

**Keywords:** femoroacetabular impingement, hip arthroscopy, long‐term outcomes, osteoarthritis, patient‐reported outcomes, total hip arthroplasty

## Abstract

**Purpose:**

To evaluate the long‐term clinical and radiographic outcomes of hip arthroscopy for femoroacetabular impingement syndrome (FAIS) in patients with mild to moderate osteoarthritis (OA). The hypothesis is that patients with FAIS and mild to moderate OA would experience sustained improvements in iHOT‐12 at 10‐year follow‐up, despite natural OA progression.

**Methods:**

This prospective cohort study included 75 patients (80 hips) with FAIS and radiographic signs of mild to moderate OA (Tönnis grade 1 or 2) who underwent hip arthroscopy between November 2011 and December 2012. The International Hip Outcome Tool (iHOT‐12) was the primary outcome at a minimum of 10‐year follow‐up. Radiographic progression of OA using Tönnis classification and conversion to THA were recorded. Statistical analysis of patient‐reported outcome measures (PROMs) was performed with Wilcoxon signed‐rank test. Relative risk assessment (RR) for conversion to THA for Tönnis grade 1 and 2 was reported.

**Results:**

At 10‐year follow‐up, 26 patients (29 hips) had undergone THA, resulting in a hip survivorship of 59% while 41% of hips progressed to THA by 10 years. The mean time to THA was 7.1 years (±1.5). Patients with Tönnis grade 2 at baseline had a significantly higher risk of THA compared with Tönnis grade 1 (RR = 3.44, 95% CI: 1.81–6.55, *p* < 0.001). Among non‐THA patients, 79% reported satisfaction with surgery. The iHOT‐12 score improved from 41.4 (±17.1) preoperatively to 71.0 (±26.7) at follow‐up (*p* < 0.001), with 67% of patients exceeding the minimal important change (MIC) threshold. Radiographic progression of Tönnis grade was observed in four hips.

**Conclusion:**

Hip arthroscopy in patients with FAIS and mild to moderate OA provides substantial long‐term functional benefits for those patients not having to undergo THA. However, preoperative OA severity is a key predictor of THA conversion with nearly two‐fifths of hips requiring THA within 10 years.

**Level of Evidence:**

Level IV, case series.

**Abbreviations**
EQ‐5D: european quality of life‐5 dimensions questionnaire
EQ VAS: european quality visual analogue scale
FAIS: femoroacetabular impingement syndrome
HAGOS: copenhagen hip and groin outcome score
HSAS: hip sports activity scale
iHOT‐12: International Hip Outcome Tool
LCEA: lateral center edge angle
mHHS: modified Harris Hip Score
MIC: minimal important change
OA: osteoarthritis
PASS: patient acceptable symptomatic state
PROMs: patient‐reported outcome measures
RCTs: randomised controlled studies
RR: relative risk
SD: standard deviation
THA: total hip arthroplasty
VAS: visual analogue scale

## INTRODUCTION

Femoroacetabular impingement syndrome (FAIS) is increasingly recognised as a contributing factor to the development of early hip osteoarthritis (OA), particularly in active and younger individuals. Among its subtypes, cam morphology has shown a strong association with OA progression, with the odds ratio (OR) ranging from 2.4 to 3.7 depending on the population and cam morphology definition [1, 13, 30, 35]. Surgical treatment of FAIS with hip arthroscopy can be clinically effective and lead to improved patient reported outcomes (PROs) [3, 5, 14]. New research findings suggest that hip arthroscopy may delay OA progression in 25% of patients, offering a potential joint‐preserving benefit [26]. Despite its success, not all patients experience equally positive results. Preexisting factors, such as severity of OA before surgery, may influence differences in outcomes [6, 29]. While patients with advanced OA are rarely considered candidates for hip arthroscopy, the impact of mild to moderate OA on surgical success of hip arthroscopy remains uncertain [2].

Research focusing on individuals with mild OA has shown that results of hip arthroscopy for FAIS, while not as optimal as those without OA, are not as significantly compromised as those seen in advanced OA cases [12, 28]. Although prior studies have explored outcomes in this group, most have limited follow‐up durations, and few have assessed joint survival or functional outcomes beyond 5 years [7, 8, 10]. Differences in clinical improvement, rates of revision surgeries, or conversion to total hip arthroplasty (THA) may become more apparent over a longer follow‐up period.

To address this knowledge gap, the current study aims to present long‐term patient reported and radiological outcomes as well as conversion rates to THA in a group of patients with both FAIS and mild to moderate hip OA treated arthroscopically. The hypothesis is that an improvement in iHOT‐12 will be observed at the 10‐year follow‐up even though the natural progression of OA, both radiographically and clinically, will continue over time.

## METHODS

**Figure 1:**
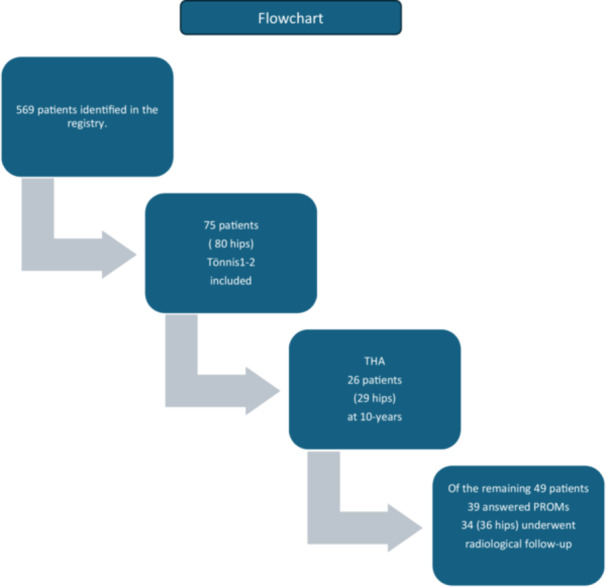
Flowchart of patients included in this study. PROMs, patient‐reported outcome measures; THA, total hip arthroplasty.

Patients with FAIS that underwent hip arthroscopy between November 2011 and December 2012 (*n* = 569) at two centres in Gothenburg, Sweden, were prospectively registered in a local hip arthroscopy registry. Preoperative radiographs were systematically evaluated for signs of OA and classified according to the Tönnis grading system [20]. Patients were eligible for inclusion if they were 18 years or older, had a confirmed diagnosis of FAIS, demonstrated radiographic signs of OA Tönnis grade 1 or 2 and had exhausted non‐operative treatment options before proceeding to surgery. The exclusion criteria were non‐primary hip arthroscopy cases (*n* = 10) and non‐FAI cases (*n* = 2). Patients with radiographic evidence of hip dysplasia, defined as LCEA < 20°, were excluded. A total of 80 hips in 75 patients (males *n* = 59, females *n* = 16) were included in the present study (Figure 1).

Demographic and perioperative information including age, sex, uni‐ or bilateral surgery, operated side, surgical and traction time was retrieved from the operative reports. All cases of conversion to THA were systematically recorded and excluded from patient‐reported outcome measures (PROMs) analysis.

Radiographs were taken preoperatively and at minimum 10‐year follow‐up. Evaluation of radiographic images was conducted by an experienced musculoskeletal radiologist. The joint space was measured at three locations on an anteroposterior (AP) pelvic radiograph (lateral sourcil, middle sourcil and above the level of the fovea). The number of hips with any joint space of 1, 2, or 3+ mm preoperatively and at 10‐year follow‐up was reported. The alpha angle on a modified Dunn projection and the lateral center‐edge angle (LCEA) on AP pelvic radiographs were measured.

The International Hip Outcome Tool (iHOT‐12) was the primary outcome measure at 10‐year follow‐up. The other PROMs used were the Copenhagen Hip and Groin Outcome Score (HAGOS, six subscales), the Hip Sports Activity Scale (HSAS), a visual analogue scale (VAS) for overall hip function, European Quality of Life–5 Dimensions Questionnaire (EQ‐5D) and European Quality of Life Visual Analogue Scale (EQ VAS) a standardised instrument evaluating health‐related quality of life as well as a single question (yes/no) reporting satisfaction after surgery [15, 16, 25, 31]. All the PROMs used, except the single question, are translated and validated in Swedish [4, 19, 25, 31].

The study was conducted with each patient giving informed consent. The Regional Ethical Review Board in Gothenburg, Sweden, granted an ethical approval for performing the study (registration number EPN 2019‐06050).

### Statistical analysis

Demographic data is presented with the use of descriptive statistics. Mean, median, standard deviation (SD), and range were used to present ordinal data. Relative and absolute frequencies were used for nominal data. To compare PROMs between preoperative and 10‐years follow‐up, non‐parametric statistical testing (Wilcoxon signed rank test) was used. The level of significance was set at *p* < 0.05. A post‐hoc power analysis showed that if 10 points of score change in iHOT‐12 is considered clinically relevant, a SD of 21 points and an *α*‐value of 0.05 a power of > 90% would be reached with 75 patients [19, 33]. Kaplan–Meier survival analysis was used to estimate hip survivorship, defined as the proportion of hips that had not undergone THA at 10‐year follow‐up. This method accounts for censored data, such as patients lost to follow‐up or those who remained THA‐free by the end of the study period. The Statistical Package for the Social Sciences (IBM SPSS statistics, version 28.0.1.1) was used to statistically analyse the patient data and PROMs.

The number of patients exceeding minimal important change (MIC) was reported with the use of a distribution‐based technique, setting the cut off value at 0.5 times the SD of the score change [23, 32]. The number of patients achieving the acceptable symptomatic state (PASS) for the six HAGOS subscales and the iHOT‐12 was also reported [18]. The PASS values are set for the iHOT‐12 63, HAGOS pain 68.8, HAGOS symptoms 62.5, HAGOS‐ADL 82.5, HAGOS‐PA 43.8, HAGOS‐Sport/Rec 60.9, and HAGOS‐QoL 42.5 [18, 19, 24].

### Surgical technique

The technique for this procedure is previously reported and is highly individualised [27]. An anterolateral portal and a mid‐anterior portal are established with the patient in a supine position. Axial traction was used to access the central compartment. The peripheral compartment was approached through a ligament‐sparing capsulotomy parallel to the fibres of the ileo‐femoral ligament and with a minimal component of transverse cut. Capsule closure was not routinely performed. In cases of pincer morphology, the bony prominence was removed with an over‐the‐top technique. The burr was placed in the peri‐labral sulcus and the resection of the pincer was made leaving the labrum in situ. In cases of larger rim resections, the labrum was re‐attached using suture anchors. The osteoplasty of the cam morphology was done thoroughly and fluoroscopy was used to assess the correction. In patients with a pistol‐grip deformity or posterior/lateral osteophytes, a resection was also performed posterior and cranial to the lateral retinacular fold to correct posterolateral impingement. Care was taken to preserve the lateral retinacular vessels. Focal cartilage lesions were treated with microfracture but not in cases of extensile cartilage loss and osteoarthritis due to its limited efficacy in diffuse degenerative changes, where the reparative capacity of the joint is significantly compromised [21, 22]. Full weight bearing was permitted postoperatively but the use of crutches was recommended for four weeks after surgery while walking outdoors. A physiotherapist supervised rehabilitation protocol was initiated directly after surgery. To decrease the risk of heterotopic ossification the patients were prescribed nonsteroidal anti‐inflammatory drugs for 4 weeks.

## RESULTS

Demographic data for the 75 patients included this study are presented in Table 1. The mean age at the time of index hip arthroscopy surgery for the THA group was 47.8 years, the percentage of male and female patients was 73% and 27% respectively and the mean follow‐up time was 130 months (±5). Out of the 80 hips included in the study, 25 hips (31%) had an isolated cam resection, and 55 hips (69%) had a combined cam and pincer resection (Table 2). At the 10‐year follow‐up, 26 patients had undergone THA and 10 were lost to follow‐up, yielding complete PROMs data for 39 of the 49 patients eligible for evaluation—corresponding to a follow‐up rate of approximately 80%.

**Table 1:** Patient demographics and perioperative data.

Total number of patients	75
Total number of hips	80
Operated side, right/left/bilateral (%)	40/53/7
Age ‐ mean (SD)	47 years (10)
Male/female (%)	77/23
Follow‐up time (SD)	130 (5) months
Symptom duration ‐ median (min–max)	48 (6–252) months
Operation time – mean (SD)	77 (18) min
Traction time – mean (SD)	11 (8) min

Abbreviation: SD, standard deviation.

**Table 2:** Arthroscopic procedures performed (80 hips).

Surgical procedure	Number of hips
Isolated CAM	25
CAM + pincer (combined)	55
Labral suture	6
Microfracture	4
Labral resection	9
Labral debridement	19
Os acetabuli	3
Ossified labrum	2
Labral calcarea	1

For the group of patients that had not undergone THA at 10‐year follow‐up, the iHOT‐12 score, which was the primary outcome, was improved from the preoperative value of 41.4 (±17.1) to 71 (±26.7) (*p* < 0.001). The percentage of patients exceeding the MIC and PASS values for iHOT‐12 was 67% and 62% respectively. All the HAGOS subscales were improved with the percentage of patients achieving the MIC and PASS values for the HAGOS‐symptoms being 77% and 64% respectively. Data regarding the PROMs values for the non‐THA patients are presented in Table 3.

**Table 3:** Outcome data.

PROMs	Preoperative mean (SD)	10‐year follow‐up mean (SD)	Change mean (SD)	Preoperative median	10 Years median	Change median (range)	% patients MIC	% Exceeding PASS	*p* value
iHOT‐12	41.4 (17.1)	71 (26.7)	29.6 (31.3)	42.9	83.7	31.7 (−33.5 to 95.8)	67	62	<0.001
HAGOS ‐Symptoms	47.4 (19.5)	70.7 (24.7)	23.3 (25.2)	42.9	75	21.4 (−32.1 to 71.4)	77	64	<0.001
HAGOS‐Pain	56.0 (17.8)	77.2 (21.4)	21.2 (24.3)	55	82.5	20 (−27.5 to 82.5)	64	64	<0.001
HAGOS‐ Daily activity	55.6 (22)	71.8 (29.6)	16.2 (34.1)	52.5	88	13.8 (−66.2 to 100)	43	52	<0.001
HAGOS Sport	40.8 (20.9)	66 (29.4)	25.2 (30.6)	34.4	78.1	25 (−34.4 to 84.4)	56	62	<0.001
HAGOS‐ Physical Activity	34.2 (27.5)	66.3 (31.7)	32.1 (40.1)	31.3	75	25 (−50 to 100)	64	69	<0.001
HAGOS‐ Quality of life	30.5 (15)	65.6 (27.6)	35.1 (30.5)	30	70	35 (−66 to 99)	69	83	<0.001
EQ‐5D‐TTO	0.8239 (0.1036)	0.8937 (0.1294)	0.0698 (0.1653)	0.8683	0.9349	0.0897 (−0.3798 to 0.3909)	NA	NA	<0.001
EQ VAS	69.7 (13.9)	76.1 (13.5)	6.4 (18)	75	79	3.5 (−26 to 61)	NA	NA	0.06
HSAS	2.3 (2.0)	3.0 (1.6)	0.7 (2.1)	2	3	1 (−6 to 4)	NA	NA	0.04
VAS‐ overall hip function	48.7 (22)	70.6 (24.8)	21.9 (30.1)	50	78	20 (−40 to 80)	NA	NA	<0.001
Satisfied with surgery %		79							

Abbreviations: EQ‐5D, EuroQoL‐5 Dimension Questionnaire; HAGOS, copenhagen hip and groin outcome score; HSAS, hip sports activity scale; iHOT‐12, international hip outcome tool; MIC, minimal important change; PASS, patient acceptable symptomatic state; VAS, visual analogue scale.

**Figure 2:**
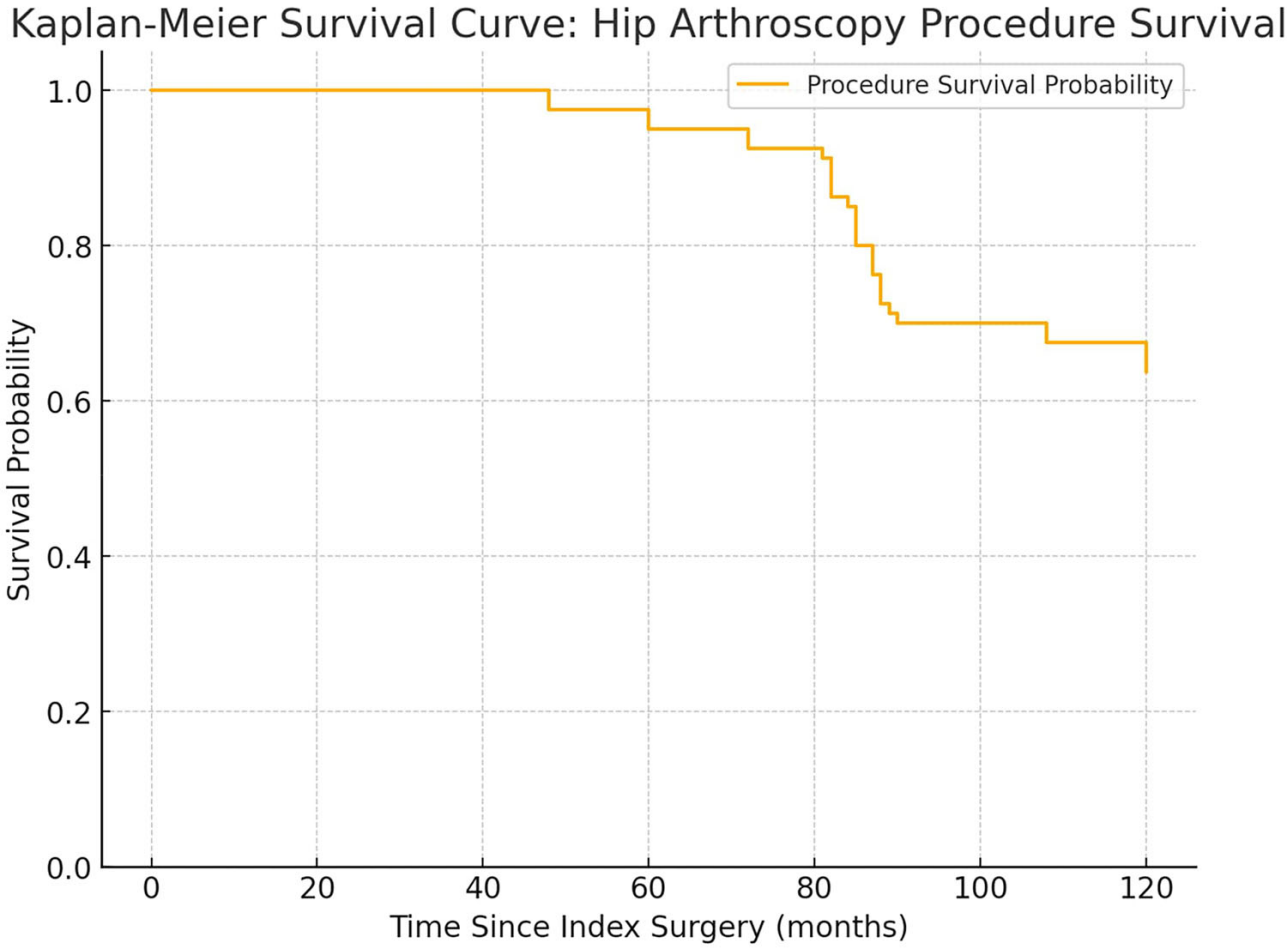
Kaplan–Meier survival curve: Hip arthroscopy procedure survival.

At 10‐years a total of 26 patients (29 hips) had undergone THA (Figure 1). The hip survivorship at 10‐years defined as the percentage of hips that were not treated with THA was 59%, while 41% progressed to THA. The mean time to THA was 7.1 years (±1.5). A Kaplan–Meier method survival curve is illustrated in Figure 2. In the group of patients that were not operated with THA (*n* = 39) 79% reported being satisfied with their surgery.

Of the 29 patients who underwent THA, 20 of the 32 patients initially classified with Tönnis grade 2 OA and 9 of the 48 with Tönnis grade 1 OA proceeded to arthroplasty following hip arthroscopy. At the 10‐year follow‐up, patients with Tönnis grade 2 at baseline had a significantly higher risk of undergoing THA compared to those with Tönnis grade 1 (RR = 3.44, 95% CI: 1.81–6.55, *p* < 0.001). This indicates that patients with greater initial hip degeneration were over three times more likely to require THA within 10 years. The method survivorship at 10‐years for Tönnis grade 1 was 81% and for Tönnis grade 2 was 38%.

In three hips (6%) in the non‐THA group a radiographic progression was observed from Tönnis grade 1 to Tönnis grade 3 and in one (3%) from Tönnis grade 2 to Tönnis grade 3. The lateral sourcil joint space was reduced from a mean of 3.6 mm (±0.9) to a mean of 3.2 mm (±1.2) (*p* < 0.05). At 10‐year follow‐up, the mean alpha angle and LCEA angle were 54° (±15) and 36° (±8), respectively, compared to preoperative values of 74° (±12) and 32° (±7). The radiographic data both preoperatively and at 10‐year follow‐up are presented in Table 4.

**Table 4:** Radiological measurements.

Measurement	Preoperatively 80 hips	10‐year follow‐up 36 hips
Alpha angle (degrees)	74 (SD 12)	54 (SD 15)
Lateral CE Angle (degrees)	32 (SD 7)	36 (SD 8)
Tönnis grade 1 (No hips)	48 (60%)	25 (69%)
Tönnis grade 2 (No hips)	32 (40%)	7 (19%)
Tönnis grade 3 (No hips)	0	4 (11%)
Any joint space of 1 (mm) (No hips)	3 (4%)	4 (11%)
Any joint space of 2 (mm) (No hips)	20 (25%)	9 (25%)
Any joint space of 3+ (mm) (No hips)	57 (71%)	23 (64%)
THA with Tönnis grade 1 preoperatively (No)		9 (31%)
THA with Tönnis grade 2 preoperatively (No)		20 (69%)

Abbreviations: CE, center‐edge; SD, standard deviation; THA, total hip arthroplasty.

## DISCUSSION

The most important finding of this study is that in this group of patients with FAIS and Tönnis grade 1 or 2 treated with hip arthroscopy, only 59% of hips survived without needing THA at 10 years. However, for the patients who were not operated with THA at the final follow‐up, the satisfaction rate was high and good PROMs were reported.

The results of this study suggest that baseline grade of OA is a key predictor of long‐term THA conversion following hip arthroscopy for FAIS. These findings are consistent with previous studies that have reported an association between preoperative cartilage degeneration and the likelihood of conversion to THA [5, 11, 34]. Chandrasekaran et al. [7] conducted a matched‐pair cohort analysis evaluating the outcomes of hip arthroscopy in patients with Tönnis grade 2 OA using control groups with Tönnis grade 0 and grade 1 OA with a two‐year follow‐up. The study concluded that the Tönnis grade 2 group had a higher risk for conversion to THA, yet no significant difference was found regarding PROMs and patient satisfaction among the groups.

In a retrospective comparative case series, the authors reported on patients who underwent primary hip arthroscopy for FAIS between 2012 and 2013, with a minimum 10‐year follow‐up [6]. Patients who were reoperated were propensity matched 1:4 to non‐reoperation patients, and groups were compared on demographics, radiographic findings, intraoperative details, and patient‐reported outcomes. Patients with more severe cartilage defects in the acetabulum and femoral head were significantly more likely to require secondary surgery following primary hip arthroscopy for FAIS. The study found that 82% of patients who converted to THA had high‐grade cartilage defects, compared to 14% of those who had not undergone revision surgery at 10 years. These findings are in concordance with findings of our study.

From a clinical perspective, these findings offer important real‐world evidence that can help inform the ongoing debate regarding optimal management strategies for patients with FAIS and moderate osteoarthritis. The high rate of conversion to THA observed in this cohort underscores the notion that patients with Tönnis grade 2 OA may represent a borderline indication for hip arthroscopy. While this study focused on a well‐defined cohort, the findings may be generalisable to other patient populations with similar FAIS presentations and early‐stage OA, particularly when using comparable surgical techniques and follow‐up protocols. This raises the question of whether alternative treatment strategies, such as early joint preservation approaches or even direct consideration of THA in select cases, should be explored for patients with moderate pre‐existing osteoarthritis. An international Delphi study performed by an expert panel comprised of 27 members from 18 countries concluded that there is clinical equipoise in terms of the best management strategy for patients with FAIS and Tönnis grade 2, and hence a randomised controlled trial for this cohort of patients is warranted [2].

In the current study substantial improvements in PROMs for those who did not undergo THA were demonstrated. These improvements align with existing literature suggesting that well‐selected patients can report good outcomes from hip arthroscopy despite the presence of early‐stage OA. In a study with 292 hips and minimum 5‐year follow‐up, 85 hips with Tönnis grade 1 were evaluated and reported significant improvements in all PROMs [10]. The survivorship rate with respect to conversion to THA for the Tönnis grade 1 group was 69% at 5 years, while in the Tönnis grade 0 group, it was 88%. No significant differences existed between preoperative or postoperative scores or survivorship between the groups.

The strengths of this study are the long follow‐up period of 10‐years as well as the high follow‐up rate of 80%. Moreover, the combination of PROMs and radiographic examinations gives a more complete understanding of the long‐term results for this patient group.

However, there are certain limitations to this study. While this is a register‐based study there is no control group that could allow a direct comparison of the PROMs and radiographic parameters as well as differences in THA conversion rates. Future studies incorporating randomised controlled trials or comparative cohort analyses would provide a more comprehensive understanding of treatment efficacy in this patient population.

Second, the sample size, while adequate for detecting significant differences, remains relatively modest. Larger multicenter studies with greater statistical power would further validate the findings in the present study and allow for more granular subgroup analyses. Another limitation of this study is that all radiographic assessments were performed by a single radiologist, which prevents the evaluation of interrater reliability and may introduce observer bias. However, previous studies have shown fair interrater reliability for these measurements [17]. Furthermore, while well‐established PROMs were used, subjective patient‐reported outcomes may still introduce a degree of bias, particularly in a long‐term follow‐up setting [9, 36].

## CONCLUSION

Hip arthroscopy in patients with FAIS and mild to moderate OA provides substantial long‐term functional benefits for those patients not having to undergo THA. However, preoperative OA severity is a key predictor of THA conversion with nearly two‐fifths of hips requiring THA within 10 years.

## AUTHOR CONTRIBUTIONS

All authors contributed to the study conception and design. Material preparation, data collection and analysis were performed by Sarantos Nikou and Carl Sandlund. The first draft of the manuscript was written by Sarantos Nikou and all authors commented on previous versions of the manuscript. All authors read and approved the final manuscript.

## CONFLICT OF INTEREST STATEMENT

The authors declare no conflicts of interest.

## ETHICS STATEMENT

The study was approved by the Swedish Ethical Review Authority. All the procedures being performed were part of the routine care. Informed consent was obtained from all individual participants included in the study.

## Data Availability

Derived data supporting the findings of this study are available from the corresponding author on request.
